# The COVID‐19 Menace

**DOI:** 10.1002/gch2.202100004

**Published:** 2021-05-07

**Authors:** Piotr Walczak, Miroslaw Janowski

**Affiliations:** ^1^ Center for Advanced Imaging Research Department of Diagnostic Radiology and Nuclear Medicine University of Maryland Marlene and Stewart Greenebaum Comprehensive Cancer Center University of Maryland Baltimore MD 21201 USA

**Keywords:** COVID‐19, drugs, mortality, SARS‐CoV‐2, transmission, vaccines, viruses

## Abstract

Coronavirus disease 2019 (COVID‐19) is caused by the new severe acute respiratory syndrome coronavirus 2 (SARS‐CoV‐2), which binds to ectoenzyme angiotensin‐converting enzyme 2. It is very contagious and is spreading rapidly around the world. Until now, coronaviruses have mainly been associated with the aerodigestive tract due to the presence of a monobasic cleavage site for the resident transmembrane serine protease 2. Notably, SARS‐CoV‐2 is equipped with a second, polybasic cleavage site for the ubiquitous furin protease, which may determine the widespread tissue tropism. Furthermore, the terminal sequence of the furin‐cleaved spike protein also binds to neuropilin receptors. Clinically, there is enormous variability in the severity of the disease. Severe consequences are seen in a relatively small number of patients, most show moderate symptoms, but asymptomatic cases, especially among young people, drive disease spread. Unfortunately, the number of local infections can quickly build up, causing disease outbreaks suddenly exhausting health services’ capacity. Therefore, COVID‐19 is dangerous and unpredictable and has become the most serious threat for generations. Here, the latest research on COVID‐19 is summarized, including its spread, testing methods, organ‐specific complications, the role of comorbidities, long‐term consequences, mortality, as well as a new hope for immunity, drugs, and vaccines.

## Graphical abstract

The enormously contagious severe acute respiratory syndrome coronavirus 2 has rapidly spread worldwide. The virus has acquired a new proteolytic site for a ubiquitous furin protease, as well as new binding site for an adhesion molecule in the brain and immune cells. In this way, the virus has a broad firepower across all organs.

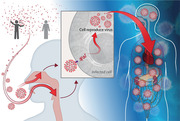

## 1 Introduction

The pandemic of the century has just swept the world unawares and turned 2020 into a nightmare. The rapid spread and high potency of the severe acute respiratory syndrome coronavirus 2 (SARS‐CoV‐2) virus contributed to the global lockdown. Industry, schools, and commuting suddenly froze. The viral outbreaks in Wuhan (China), Lombardia (Italy), and New York (USA) have not left the headlines for weeks and have become the collective experience of a generation. Subsequently, coronavirus disease 2019 (COVID‐19) reached nearly every corner of the earth. Alarming mortality rates of 1–5% (10× more than the flu) and lingering symptoms complement the COVID‐19 menace. It is related to the widespread distribution of not only SARS‐CoV‐2 receptor angiotensin‐converting enzyme 2 (ACE2), but also an acquisition of a new cleavage site accessible to ubiquitously present furin enzyme, which enables viral infection in majority of tissues.

## 2 The Mystery of the Spread of SARS‐CoV‐2

The rapid spread of the virus worldwide and considerable variations in the disease's course and its severity are at the root of the present tragedy. Therefore, it is natural to look at the contagiousness of the virus first. This process consists of three stages: primary contact with the pathogen, subsequent spread in the body and amplification, and finally discharge of infectious virions to the environment. Detailed knowledge of these processes is critical to taking appropriate actions to contain a pandemic. The way we detect the virus and its pathomechanism can also help understand the biology of the virus and prevent its spread.

### 2.1 Transmission Mechanics

There are several mechanisms for the transmission of SARS‐CoV‐2, but the main route is through aerosolized droplets produced by the mucous membranes and saliva.^[^ ^1^ ^]^ It has been shown that microdroplets can travel at least 1–2 m in still air and up to 6.6 m under winds of 2 m s^−1^, regardless of the air humidity.^[^ ^2^ ^]^ Then, the droplets pass directly or through contact with the contaminated surface to the respiratory and digestive systems’ mucous membranes, thus initiating infection in the new subject. Notably, the nasal mucosa is the primary material tested for the virus's presence, although this route may be of minor importance for the discharge of infectious virions. What is more, this testing strategy may even lead to confusing information about the process of spreading the disease. Asymptomatic colonization of the nasal mucosa by SARS‐CoV‐2 results in an inadequate immune response (40% seroreversion in the early convalescent phase compared to 12% in symptomatic patients). This scenario is associated with the prolonged presence of the transmission‐potent virus in asymptomatic patients (19 days) compared to symptomatic infection (14 days).^[^ ^3^ ^]^ The presence of the virus in saliva may be a better predictor of a person's contagiousness as it indicates the virus's entry into the salivary glands, i.e., its systemic spread. Although nasal swabs are considered generally safe, improper testing may even be a traumatic procedure, with as serious consequences as leakage of the cerebrospinal fluid; the nasal injury may also facilitate the virus's systemic spread.^[^ ^4^ ^]^

It is noteworthy that several over‐the‐counter mouthwash/rinse products, including Listerine and Listerine‐like products, were highly effective at inactivating infectious virus with over 99.9% efficacy even with a 30 s contact time.^[^ ^5^ ^]^ Thus, mouthwashes may also be important in inhibiting the spread of COVID‐19 by killing viral particles in saliva.^[^ ^6^ ^]^ Though, SARS‐CoV‐2 replication sites are not limited to oral mucosa, thus antiviral effects of mouthwashes are most likely short‐lasting. Especially, since SARS‐CoV‐2 has been shown to remain infectious for a much longer period than was generally believed to be possible (28 days at 20 °C and 24 h at 40 °C).^[^ ^7^ ^]^ The survival time of SARS‐CoV‐2 on the human skin was ≈9 h, much longer than that of the Influenza A virus (≈1.8 h). The longer survival of SARS‐CoV‐2 on the skin increases contact transmission risk; however, hand hygiene can reduce this risk.^[^ ^8^ ^]^ Therefore, detecting a virus in saliva and its inactivation in the oral cavity may be more critical from a clinical point of view. In summary, improving knowledge of virus transmission mechanisms is essential for developing appropriate epidemiological policy and effective pandemic containment.

### 2.2 Population‐Level Investigations

Understanding the spread of the virus at the level of populations is another critical factor in the effective management of a pandemic. Contact tracing is an essential tool to achieve this objective.^[^ ^9^ ^]^ Especially, since there is a probability that 40% of all positive cases are asymptomatic.^[^ ^10^ ^]^ This statement has also been supported by analyzing the COVID‐19 outbreak on the aircraft carrier.^[^ ^11^ ^]^ Population‐level investigations can also be very informative for policymaking and ensuring rapid responses to emerging outbreaks. A prime example is an Indian study conducted in Tamil Nadu and Andhra Pradesh, which revealed a high prevalence of SARS‐CoV‐2 infections in children and adults having contact with them. That study pointed to schools as an important medium of SARS‐CoV‐2 transmission, providing the basis for critical decisions at the level of society, such as cancellation of in‐person classes and transferring educational activities to the virtual space.^[^ ^12^ ^]^ The internet's enormous power has been used for the early identification of COVID‐19 hotspots by detecting increased online search for keywords related to gastrointestinal symptoms.^[^ ^13^ ^]^ Wastewater monitoring proved to be another powerful tool in tracking the spread of COVID‐19.^[^ ^14^ ^]^ Population‐level genomic studies can be another important instrument to understand the prevalence of genetic variants, which may be linked with decreased recognition of the neutralizing antibody.^[^ ^15^ ^]^ Another study identified a population of patients with prolonged presence of a virus, as evidenced by positive results of nasal swabs despite completed recovery after infection, and this phenomenon was observed at the worrying rate of 16.7% patients.^[^ ^16^ ^]^ However, it is not yet possible to fully understand the prolonged presence of the virus for infectivity in patients and the possibility of persistent symptoms.

Population studies on the prevalence of immunity against SARS‐CoV‐2 components are also critical to understanding disease spread. It has been shown that immunoglobulin G (IgG) immunity targeting the S2 subunit is prevalent in uninfected children and adolescents. In turn, people infected with COVID‐19 have concomitant immunoglobulin M (IgM) and immunoglobulin A (IgA) antibodies against both S1 and S2 subunits. This phenomenon may be responsible for a milder or asymptomatic course of SARS‐CoV‐2 infection in this population.^[^ ^17^ ^]^ Cellular immunity analysis revealed that 40–60% of unexposed individuals and 100% of COVID‐19 convalescents had CD4+ T lymphocytes reactive to SARS‐CoV‐2. It points to cross‐reactivity with “common cold” coronaviruses, as well as the important role of the cellular anti‐COVID‐19 response. An independent study shows robust T‐cell immunity in convalescents with asymptomatic or mild COVID‐19.^[^ ^18^ ^]^

Population studies are also instrumental in identifying high‐risk groups who need special attention. Unfortunately, health‐care workers (HCWs) are particularly vulnerable this time around. The Minnesota Department of Health has required reporting of HCW exposure to SARS‐CoV‐2 to enroll these individuals with higher risk into quarantine and symptom monitoring. Out of 21 406 HCW exposures, 5374 (25%) were classified as higher risk, and they were enrolled in direct patient care and nonpatient care interactions. Then, a third of HCWs were involved in symptom monitoring, and half of them tested positive for COVID‐19. Overall, 7% of HCWs with higher risk tested positive for SARS‐CoV‐2. Therefore, HCWs are at increased risk for COVID‐19 as a result of workplace exposure.^[^ ^19^ ^]^ Another study conducted on the cohort of 158 445 HCWs revealed that HCWs who do not have direct contact with COVID‐19 patients directly and family members of these HCWs have a similar risk of hospitalization as the general population. On the other hand, patient‐facing HCWs have 3 times higher risk of being admitted to the hospital and twice as high for their household members. The greatest risk is for HCWs dealing with admitting patients. Overall, health‐care professionals and their households account for one‐sixth of the COVID‐19 cases admitted to hospitals.^[^ ^20^ ^]^ In conclusion, there probably has not been another disease that is so prevalent in HCWs. People with group O also have a much lower relative risk of SARS‐CoV‐2 infection, but there is no impact on hospitalization and death from COVID‐19.^[^ ^21^ ^]^ It is related to the SARS‐CoV‐2 binding to the blood antigen present on respiratory epithelial cells.^[^ ^22^ ^]^ Population studies are also relevant to understanding patient dynamics in emergency departments (E.D.s). Return hospital admissions were analyzed among 1419 COVID‐19 patients discharged from five emergency departments in the USA. The study found that ≈5% of COVID‐19 patients discharged from the E.D. returned for an unscheduled hospital admission within 72 h. Age, abnormal chest X‐rays, and fever or hypoxia on admission were independently associated with increased hospital returns. The COVID‐19 pandemic has challenged emergent care providers to undertake urgent interventions under difficult circumstances. An additional challenge is patients who appear well enough to be discharged at the first visit but may require subsequent admission. As the pandemic evolves, further investigation may be needed to develop risk stratification tools that guide the management of COVID‐19 patients in the E.D.^[^ ^23^ ^]^ There is also growing body of evidence on impact of apolipoprotein E allele on COVID‐19. It has been shown in older, Spanish population that ε4 variant of the APOE gene is associated with the presence and clinical severity of coronavirus infection.^[^ ^24^ ^]^ In the UK biobank cohort, the ε4 variant was associated with increased risk of test positivity and of mortality with test‐confirmed COVID‐19.^[^ ^25^ ^]^

Much effort has also gone into modeling and predicting disease progression. A retrospective cohort of 832 consecutive admissions for COVID‐19 from March 4 to April 24, 2020, with follow‐up through June 27, 2020, to five hospitals in MD and Washington, DC, was subjected to analysis. The patient's likelihood of disease progression varied significantly with age, nursing home residence, comorbid conditions, obesity, respiratory symptoms, respiratory rate, fever, absolute lymphocyte count, hypoalbuminemia, troponin level, C‐reactive protein level, and the interaction between these factors. The factors present on admission were used to establish a model predicting in‐hospital disease progression with an area under the curve of 0.85, 0.79, and 0.79 at days 2, 4, and 7 of hospitalization, respectively.^[^ ^26^ ^]^

Increased alcohol consumption during lockdowns in the USA is proven by a 54% increase in domestic alcohol sales for the week ending March 21, 2020, compared to a year earlier. Online sales increased by 262% from 2019. A self‐reporting study revealed an increase in alcohol consumption of up to 20% in the various groups surveyed. This should be considered because, in addition to a number of negative physical health associations, excessive alcohol use may lead to or worsen existing mental health problems, such as anxiety or depression, which may worsen during COVID‐19.^[^ ^27^ ^]^

### 2.3 Molecular Mechanisms of Infectivity

Viruses hijack cellular pieces of machinery to multiply and spread in organisms. Surface molecules serve as viral gateways. The structural similarity to its predecessor SARS‐CoV‐1 quickly put the scientists on the right track of ACE2 as a viral target. ACE2 is an ectoenzyme that is abundant on the surface of pulmonary endothelium^[^ ^28^ ^]^ and olfactory neuroepithelium.^[^ ^29^ ^]^ It is also scattered throughout the body, including a smaller germinal form expressed exclusively in the testes. ACE2 regulates blood pressure, electrolyte balance, sympathetic activity, and other essential functions.^[^ ^30^ ^]^ However, it should be emphasized that there are significant differences in the clinical presentation of SARS‐CoV‐1 and Middle East Respiratory Syndrome (MERS), as opposed to SARS‐CoV‐2. The former mainly targeted the lungs, while the latter has a much more comprehensive portfolio of target organs, including very alarming anosmia and lack of taste, which points to the central nervous system (CNS). Therefore, further research is being conducted to reveal potential surface targets. Heparan sulfate (H.S.) has been shown to serve as a necessary host attachment factor promoting SARS‐CoV‐2 infection of various target cells. However, it is known that the glycocalyx is the first point of contact for all pathogens that infect the cells of mammals. Thus, it is not surprising that many viruses exploit glycans such as H.S. as attachment factors. Therefore, this target cannot rather be considered as responsible for specific pathomechanism of SARS‐CoV‐2. However, it can still be considered as a therapeutic target.^[^ ^31^ ^]^

**Figure 1:**
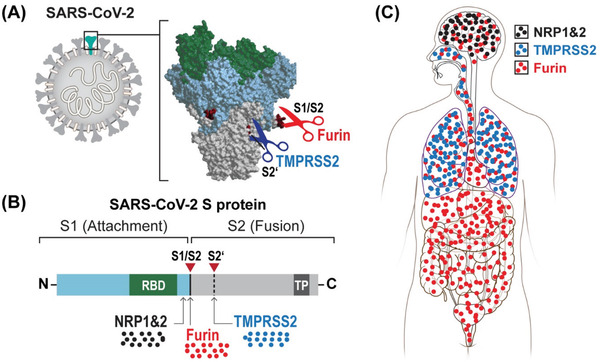
A) Proteolytic and B) binding sites of SARS‐CoV‐2 spike protein and C) biodistribution in the body depending on them. S1 – segment 1 of spike protein, S2 – segment 2 of spike protein, RBD – receptor binding domain to angiotensin‐converting enzyme 2 (ACE2), TMPRSS2 – transmembrane protease, serine 2, NRP – neuropilin, TP – transmembrane protein.

SARS‐CoV‐1 and MERS viruses use the spike (S) protein's monobasic cleavage by the TMPRSS2 protease, which occurs predominantly in the aerodigestive tract.^[^ ^32^ ^]^ Importantly, SARS‐CoV‐2 can also undergo furin‐mediated cleavage of the spike (S) protein at an additional S1/S2 site (**Figure** **1**). The ubiquitous furin expression may determine the widespread tissue tropism of SARS‐CoV‐2 distinct from its ancestor coronaviruses.^[^ ^33^ ^]^

Also, a closer look into furin‐mediated cleavage of the spike (S) viral protein revealed the production of a polybasic C‐terminal sequence Arg—Arg—Ala—Arg on the S1 polypeptide, which conforms to a C‐end rule (CendR) motif that binds to neuropilin‐1 (NRP1) and neuropilin‐2 (NRP2) receptors on the cell surface. Subsequent X‐ray crystallography and biochemical studies confirmed that the S1 CendR motif directly binds NRP1. Moreover, blocking this interaction using ribonucleic acid interference or selective inhibitors reduced SARS‐CoV‐2 entry and infectivity in cell culture. Thus, NRP1 serves as a host factor for SARS‐CoV‐2 infection and can be a therapeutic target for COVID‐19. The affinity for neuropilin receptors could potentially contribute to explaining the extensive involvement of the CNS in the COVID‐19 course.^[^ ^34^ ^]^

It has been shown not only on a population level but also molecular studies using iPS‐derived brain cells revealed increased rate of SARS‐CoV‐2 infection in APOE ε4 variant neurons and astrocytes.^[^ ^35^ ^]^

In summary, the acquisition of the S1/S2 cleavage site by SARS‐CoV‐2 might be essential for understanding pathological features distinct from other coronaviruses. Thus, targeting this cleavage site may be pivotal for effective pre‐ and postexposure therapeutic interventions.

## 3 Detection of SARS‐CoV‐2 Infection

**Figure 2:**
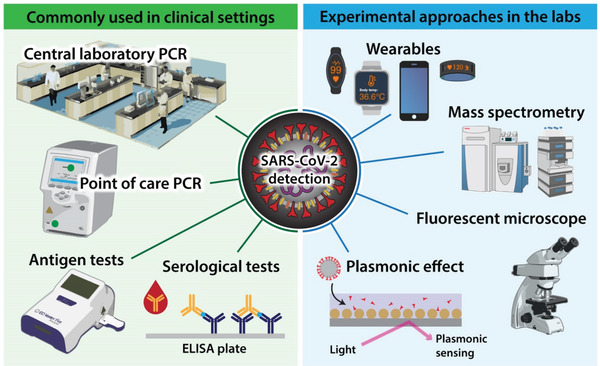
Diagnostic opportunities for SARS‐CoV‐2. PCR – polymerase chain reaction, ELISA – enzyme‐linked immunosorbent assay.

An adequate method of virus detection is essential to containing the COVID‐19 pandemic effectively and there are various approaches available (**Figure** **2**). Polymerase chain reaction (PCR) performed in centralized laboratories is the gold standard for SARS‐CoV‐2 detection due to its high sensitivity and specificity. This method also informs about the genomic load, which correlates with worse outcomes, including the risk of death.^[^ ^36^ ^]^ However, this approach is expensive and slow and, therefore, cumbersome for the clinical workflows. As such, work is underway to improve SARS‐CoV‐2 testing.

Point‐of‐care PCR testing using QIAstat‐Dx respiratory SARS‐CoV‐2 panel drastically reduced the time to obtain results (1.7 h). It can help improve infection control and patient flow compared to centralized laboratory PCR testing (21.3 h) without sacrificing quality. These results were generated by the University Hospitals Southampton NHS Foundation Trust, and they show the feasibility of point‐of‐care PCR testing to improve the speed but not cost.^[^ ^37^ ^]^

Antigen detection is a fast and low‐cost approach to testing for SARS‐CoV‐2. Analysis of samples collected at a public site testing in San Francisco revealed that of the 878 subjects tested by reverse transcriptase‐PCR, 3% (26/878) were positive, of which 15/26 had a cycle threshold (Ct) < 30, indicating high viral load. Using the Ct < 30 cutoffs for evaluation of the Binax‐CoV‐2 antigen detection assay, the sensitivity of Binax‐CoV‐2 was found to be 93.3% (14/15), and specificity 99.9% (862/863).^[^ ^38^ ^]^ Therefore, antigen detection assays can play a role in widespread testing to better understand the overall dynamics of COVID‐19.

Another approach, which allows rapid and low‐cost detection of viral RNA, used the plasmonic effect, and allowed the naked eye to interpret the result. Still, no sensitivity or specificity of this method was reported.^[^ ^39^ ^]^ The targeted electrochemical biosensor chip exploits the same plasmonic phenomenon and gives results rapidly but at a higher cost, with almost 100% accuracy, sensitivity, and specificity.^[^ ^40^ ^]^ The use of the same plasmonic effect to detect spike protein has also been described.^[^ ^41^ ^]^ Plasmonic biosensor designs for the detection of other viruses are advancing rapidly.^[^ ^42^ ^]^

Serological testing for SARS‐CoV‐2 is another popular approach used in the diagnosis of COVID‐19. Although, it does not detect the virus itself but rather antibodies directed against it. Therefore, this strategy is less valuable in the acute phase of the disease but has high epidemiological value. It enables the detection of seroconversion. For this purpose, the lateral flow assay testing platform is most frequently used.^[^ ^43^ ^]^ Enzyme‐linked immunosorbent assay and immunofluorescence are two other serological methods.^[^ ^44^ ^]^

There are also more experimental attempts in the diagnosis of COVID‐19. Single‐particle imaging by wide‐field fluorescent microscopy, based on the binding of short fluorescent DNA to the surface of viral particles combined with a convolutional neural network, made it possible to identify SARS‐CoV‐2 viral particles and distinguish them from other viruses within 5 min.^[^ ^45^ ^]^ Targeted mass spectrometry is also a rapid, specific (100%), and relatively sensitive (90%) method of SARS‐CoV‐2 detection, although with limited access due to the very high cost of instruments.^[^ ^46^ ^]^ There is also an effort to use wearable devices for COVID‐19 diagnosis. Data obtained from wearables (heart rate, sleep, and activity) and self‐reported symptoms revealed that the combination of symptom data and wearable sensor data resulted in an area under the curve (AUC) of 0.80 to discriminate symptomatic individuals with COVID‐19 from negative individuals, a significantly better performance (*p* < 0.01) than a model that considers symptoms alone (AUC = 0.71). Such continuous passively captured data can complement virus testing, which is generally a one‐off or infrequent sampling assay.^[^ ^47^ ^]^

Cell culture has been used for decades for virus isolation and identification.^[^ ^48^ ^]^ However, this method is slow and it requires considerable technical expertise. Therefore, it is not used for diagnosis of COVID‐19 patients, but this method has rather role in the research on the infectivity of specimens.^[^ ^49^ ^]^

## 4 Organ Involvement

### 4.1 Lungs

Pneumonia with an abnormal chest computer tomography became the flagship symptom of the Wuhan outbreak, which then turned into the COVID‐19 pandemic.^[^ ^50^ ^]^ It quickly became obvious that it is a severe, even fatal, respiratory disease in which nearly half of the patients experience dyspnea.^[^ ^51^ ^]^ Hence, the great interest in lung pathology is a key to understanding the pathomechanism leading to lung injury.

A systematic study of consecutive 41 postmortem samples from COVID‐19 patients revealed extensive alveolar damage (41/41 of patients) and both micro‐ and macrovascular lung thrombosis (29/41, 71%). Pneumocytes and endothelial cells contained viral RNA even in the later stages of the disease. There was also the frequent presence of many dysmorphic pneumocytes, often forming syncytial elements (36/41, 87%). In other organs of these patients, no obvious signs of viral infection were detected.^[^ ^52^ ^]^

In patients who died from COVID‐19‐associated or influenza‐associated respiratory failure, peripheral lung histology revealed diffuse alveolar damage with perivascular T‐cell infiltration. Lungs of COVID‐19 patients also showed distinctive vascular features, consisting of severe endothelial injury associated with an intracellular virus and disrupted cell membranes. Histologic pulmonary vascular analysis in patients with COVID‐19 has shown widespread thrombosis with microangiopathy. The alveolar‐capillary microthrombi were 9 times as prevalent in patients with COVID‐19 as in patients with influenza (*p* < 0.001). In the lungs from patients with COVID‐19, the amount of new vessel growth – predominantly through the mechanism of intussusceptive angiogenesis – was 2.7 times of that in the lungs from patients with influenza (*p* < 0.001).^[^ ^53^ ^]^

Another study revealed that hyaluronan is a substance that accumulates in the alveoli and obstructs the airways. As such, hyaluronan may be an attractive target for the inhaled delivery of degrading enzymes.^[^ ^54^ ^]^

In summary, there is a high probability that the severity of the disease may be due to an unusual pathomechanism. The lungs appear to be attacked from both sides: from the outside world through the alveoli and internally through the pulmonary vessels. The inhalation route of infection contributes to the involvement and damage of pneumocytes, which is already damaging. However, once the virus breaks the mucosal barrier and reaches the bloodstream, it may preferentially return to the lungs due to the abundance of ACE2 on the intraluminal surface of endothelial cells. In this respect, it is somewhat similar to SARS‐CoV‐1, which is known to have a high incidence of deadly pneumonia. However, there are also differences in lung involvement as SARS and MERS are rather unilateral diseases, while COVID‐19 is characterized by a relatively symmetrical pathology.^[^ ^55^ ^]^ These features of COVID‐19 disease may be associated with the higher stability of SARS‐CoV‐2, which may use the gastrointestinal gateway more frequently and then attack the lungs symmetrically from the vascular side. The abovementioned additional furin‐mediated cleavage site may increase the risk of lung and other organ involvement. Therefore, if the lungs are attacked from only one side (vascular or alveolar), the patient may have a better prognosis. At the same time, massive lung attack from both sides is likely too severe and leads to death in many instances.

### 4.2 Cardiovascular System

The above description of pulmonary pathologies emphasizes the important role of the circulatory system in viral morbidity. Major thromboembolic and cardiovascular events occurred with a high frequency (double‐digit 30–40%) in patients requiring an intensive care unit (ICU) stay. By contrast, thromboembolic complications in non‐ICU hospitalizations were one order of magnitude less frequent at single‐digit numbers. No such events have been reported in outpatient treatment, but their occurrence may indicate hospital admission. It is worth noting that other heart‐related conditions were also much more common, such as heart failure, myocarditis, and new atrial fibrillation. Acute respiratory distress syndrome revealed the strongest association with thromboembolic and cardiovascular complications.^[^ ^56^ ^]^ Hypercoagulability was also demonstrated in another study comparing the results of vascular imaging in patients with COVID‐19 (11%) and control patients noninfected with SARS‐CoV‐2 (1% of the investigated population).^[^ ^57^ ^]^

Viral pathology also covers the heart itself. Cardiac magnetic resonance (MR) of 100 unselected patients who recently recovered from COVID‐19 (1/3 hospitalized) revealed heart involvement in 78% and ongoing cardiac inflammation in 60% of participants. Endomyocardial biopsy in severely symptomatic patients revealed lymphocytic inflammation. Troponin was also detected in 71% of patients and significantly elevated by 5%. Compared to healthy controls and a matched risk factor control group, patients that recovered from COVID‐19 had a low left ventricular ejection fraction, greater left ventricle volume, higher left ventricle mass, and raised native T1 and T2. There was little difference in native T1, but not in T2 mapping between hospitalized and nonhospitalized cases.^[^ ^58^ ^]^

The cardiac dysfunction manifested by reduced contractility and elevated circulating cardiac enzymes has been observed in up to 50% of patients. The most characteristic pathological changes were myofibrillar fragmentation into individual sarcomeres and cardiomyocyte enucleation. This fragmentation's consistency indicates cleavage by a specific protease, including the possible presence of SARS‐CoV‐2 papain‐like protease (PLpro/Nsp3). However, this cytopathy was independent of active SARS‐CoV‐2 replication, but did not occur after exposure to the inactivated enzyme. Thus, the pathological mechanisms leading to COVID‐19‐induced heart damage are still unknown. It is disturbing that subclinical heart injury also occurs in mildly ill patients and may lower the cardiac reserve and accelerate the heart's aging.^[^ ^59^ ^]^ Therefore, it could profoundly impact our population on a global scale in the years to come. It also raises the need for regenerative medicine approaches to combat long‐term COVID‐19 consequences in heart pathology.

COVID‐19 is also linked to cardiac arrests. A preliminary study from China revealed that less than 1% of COVID‐19 patients resuscitated due to cardiac arrest had a favorable outcome. The first but still small American study revealed that no such patient following cardiac arrest survived to the discharge from hospital.^[^ ^60^ ^]^ However, cardiac arrest is relatively frequent in critical inpatients and occurred in 701 out of 5019 cases (14%). Cardiopulmonary resuscitation was performed in 57% of them with poor survival (21.2% of patients <45 years, and only 2.9% of >80 years). As most resuscitated patients had pulseless electrical activity or asystole, this rather indicates noncardiac causes such as respiratory dysfunction and thromboembolic events. Notably, these findings are comparable to other critical illnesses and indicate the severity of COVID‐19 rather than primary heart problems as a basis for cardiac arrest.^[^ ^61^ ^]^

Pulmonary embolism is another deadly vascular event. However, it has been shown that it is not significantly more frequent in COVID‐19 patients (11.8%) than in control patients (8.6%). D‐dimer levels were the explanatory factor for both groups (2616 ng mL^−1^ in COVID‐19 patients vs 2354 ng mL^−1^ in control patients) with an AUC 0.825 for COVID‐19 and 0.81 for control patients. These results show that COVID‐19 status did not predict pulmonary embolism and elevated D‐dimer is the greatest risk factor.^[^ ^62^ ^]^

### 4.3 Immune System

The immune system is a highly orchestrated entity designed to combat infections. Thus, any imbalance or weakening has a profound effect on the body's response to COVID‐19. Dysregulation of the immune system in the course of the infection can also redirect the effectors from fighting the virus to autoaggression and destruction of the body's vital elements. Changes in both adaptive and innate immunity and humoral and cellular arms were observed in the context of COVID‐19 infection, so the impact on the immune system is widespread.

At least half of COVID‐19 patients develop antiphospholipid autoantibodies resembling antiphospholipid syndrome. These autoantibodies were shown to promote neutrophil activity in vitro and thrombosis in a mouse model. Moreover, their higher titers are also correlated with higher platelet counts, lower clinical estimated glomerular filtration rate, and more severe respiratory disease. Therefore, the autoimmune reaction may be an important pathological factor contributing to the severity of COVID‐19.^[^ ^63^ ^]^ Another study found that autoreactivity is widespread and targets type‐I interferons and other well‐known autoantibodies, including antinuclear antibodies, antineutrophil cytoplasmic antibodies, and rheumatoid factor, and maybe a manifestation of the broader phenomenon of breaking self‐tolerance to a variety of autoantigens.^[^ ^64^ ^]^ It was also found that 10% of severely ill patients have autoantibodies to interferon, which has not been seen in patients with mild or moderate disease. Interferons are part of the antiviral response so that such antibodies can dampen the host immune response against the virus.^[^ ^65^ ^]^

There is also a report describing three cases of myasthenia gravis in the course of COVID‐19. We note that myasthenia gravis appeared within 5–7 days after the onset of fever, and the time from presumed SARS‐CoV‐2 infection to the onset of myasthenia gravis symptoms is consistent with the time from infection to symptoms in other neurologic disorders triggered by infections.^[^ ^66^ ^]^

COVID‐19 also leads to a decrease in the number of T lymphocytes (mostly CD4 and CD8), while the level of interleukin (IL)‐6, IL‐2‐R, IL‐10, and tumor necrosis factor‐α increases.^[^ ^67^ ^]^ A reduction in the percentage of blood T lymphocytes is also a predictor of COVID‐19 severity.^[^ ^68^ ^]^ A closer look at the T‐cell compartment revealed that their activation and exhaustion may be a lymphopenia source.^[^ ^69^ ^]^

Cytokine release syndrome (CRS) or cytokine storm is a well‐known life‐threatening condition associated with bacterial sepsis and chimeric antigen receptor T‐cell therapy. Four proinflammatory cytokines, IL‐6, IL‐8, monocyte chemotactic protein‐1 (MCP‐1), and IL‐10, and the coagulation cascade activator plasminogen activator inhibitor‐1 (PAI‐1) constitute the standard effector arm of the CRS. IL‐6 seems to play a supervisory role among the factors mentioned above. Plasma from severe COVID‐19 patients also showed elevated IL‐6, IL‐10, and MCP‐1, but except PAI‐1, these were lower than in the case of other causes of CRS. Altogether, this points to the role of thromboembolic events in end‐stage COVID‐19 pathology. In this context, IL‐6 blockade by tocilizumab may be a therapeutic avenue.^[^ ^70^ ^]^ Another study confirmed that CRS does not play an essential role in other typical conditions, and therefore doubts the high effectiveness of anticytokine therapies.^[^ ^71^ ^]^ The immune system activation pattern was used to calculate a linear prognostic Dublin–Boston score based on the interleukin‐6 to the interleukin‐10 ratio for predicting outcomes in COVID‐19.^[^ ^72^ ^]^

Complement dysfunction may contribute to the adverse events following SARS‐CoV‐2 infection. Finally, in a candidate‐driven genetic association study of severe SARS‐CoV‐2 disease, putative complement and coagulation‐associated loci were identified, including missense, expression quantitative trait loci, and splicing quantitative trait loci variants of the critical complement and coagulation regulators. In addition to providing evidence that complement function modulates the outcome of SARS‐CoV‐2 infection, the data point to putative transcriptional genetic markers of susceptibility to COVID‐19.^[^ ^73^ ^]^

Monocytes and macrophages have been also found to be targeted by SARS‐CoV‐2 but their infection is abortive. However, still it drives host immunparalysis facilitating COVID‐19 progression.^[^ ^74^ ^]^ It has been also demonstrated that dysregulated macrophage response can be damaging to the host,^[^ ^75^ ^]^ and it has been even coined macrophage activation syndrome in COVID‐19.^[^ ^76^ ^]^

### 4.4 Kidney

The outcomes of 3345 adults with COVID‐19 and 1265 without COVID‐19 were compared to a historical cohort of 9859 patients hospitalized a year earlier in the same extensive health system in New York. A dramatic increase in acute kidney injury (AKI) was demonstrated from 25.1% in a historical cohort to 56.9% in COVID‐19 patients. Also, COVID‐19 patients required renal replacement therapy more frequently and were less likely to recover renal function. COVID‐19 patients with and without AKI were more likely to die than their counterparts without COVID‐19.^[^ ^77^ ^]^ Another group reported 7% of AKI among 1392 patients hospitalized for COVID‐19 in a tertiary teaching hospital in Wuhan in the early phase of the pandemic with an unusually high mortality rate of 60–80%.^[^ ^78^ ^]^ Thus, COVID‐19 significantly increased AKI incidence among hospitalized patients and therefore increased the risk of death. COVID‐19 infection was also found to be a predictor of AKI development. In an international observational study of adult patients hospitalized for COVID‐19, soluble urokinase receptor (suPAR) levels were measured in 352 plasma samples of adult patients collected within 48 h of admission. These suPAR levels were predictive of in‐hospital AKI and dialysis need. SuPAR may also be a vital component of the pathophysiology of AKI in COVID‐19.^[^ ^79^ ^]^

### 4.5 Brain

Proper brain function plays a vital role in social and professional independence. Thus, brain diseases, including neurological and psychiatric conditions, are a costly burden for society.

Unlike other coronaviruses and common infections, SARS‐CoV‐2 is more frequently infecting the brain. It has been experimentally demonstrated at the level of the human brain organoids and ACE2 overexpressing mice. Brain biopsy of patients who died from COVID‐19 proved the presence of SARS‐CoV‐2 in cortical neurons.^[^ ^80^ ^]^ Molecular data have been corroborated by imaging. An analysis of 60 recovered patients and 39 age‐ and sex‐matched controls hospitalized in January and February, followed up by a MRI assessment in May 2020, revealed that 55% of patients had some neurological or psychiatric symptoms at this time. MR findings revealed possible disruption to the brain's microstructural and functional integrity during the recovery stages of COVID‐19, indicating a possibility of the long‐term consequences of SARS‐CoV‐2.^[^ ^81^ ^]^

There have also recently been three case reports of Parkinson's disease (P.D.) first identified within weeks of being diagnosed with COVID‐19, and pathophysiological features are rather alarming. In all cases, brain imaging revealed the reduced function of the nigrostriatal dopamine system, akin to P.D. None of the patients had prodromal symptoms, which rather points to COVID‐19 as the cause of P.D. However, it is possible that these patients had a lower nigrostriatal reserve and would ultimately develop P.D., especially since they were all relatively young for this disease. There could be three explanations for this phenomenon: vascular damage due to a hypercoagulative state, neuroinflammation induced by marked systemic inflammation, and the direct neurotropic character of SARS‐CoV‐2. There may even be some common properties of COVID‐19 and P.D., such as hyposmia and dysgeusia, so viral spread from the nasal cavity to the nigrostriatal system is probable. Moreover, midbrain dopamine neurons express high levels of ACE2. Since α‐synuclein is a viral restriction factor, it may also be induced by COVID‐19. Its sustained elevated levels may lead to aggregates’ formation resembling P.D. and induction of dopaminergic cell death. Therefore, in the wake of the COVID‐19 pandemic, there is a risk of initiation of neurodegenerative changes and raising the frequency of P.D. in the future.^[^ ^82^ ^]^

COVID‐19 also appears to affect cognitive function. Analysis of cognitive test data from 84 285 participants of the Great British Intelligence Test demonstrated that patients who have recovered from COVID‐19 still have significant cognitive deficits, even when controlling for confounding factors. Moreover, the substantial effect size was observed not only for hospitalized by also for mild COVID‐19 cases. However, longer studies are needed to look into this phenomenon's dynamics should these deficits subside or rather increase over time. Overall, COVID‐19 can profoundly impact societies and create gaps between the high demands of intellectual work and potentially blunted intelligence of COVID‐19 convalescents.^[^ ^83^ ^]^

A prospective, multicenter observational study of 4491 consecutive hospitalized COVID‐19 patients in the New York City metropolitan area revealed new neurological disorders in 13.5% of patients. Common diagnoses were: toxic/metabolic encephalopathy (6.8%), seizure (1.6%), stroke (1.9%), and hypoxic/ischemic injury (1.4%). No patient had meningitis/encephalitis or myelopathy/myelitis, and none of the 18 cerebrospinal fluid specimens tested positive for SARS‐CoV‐2. Patients with neurological disorders frequently suffered from other comorbidities as well. After adjusting for confounding factors, COVID‐19 patients with neurological disorders had an increased risk of in‐hospital mortality (hazard ratio 1.38, *p* < 0.001).^[^ ^84^ ^]^

There is also strong evidence that COVID‐19 induces psychiatric conditions. TriNetX Analytics Network, a global federated network that captures anonymized data from electronic health records in 54 U.S. health‐care organizations, totaling 69.8 million patients, included 62 354 patients diagnosed with COVID‐19 between Jan 20, and Aug 1, 2020. In patients with no previous psychiatric history, the diagnosis of COVID‐19 was associated with an increased incidence of first psychiatric diagnosis in the following 14–90 days post COVID‐19 compared with six other health events such as influenza, other respiratory tract infections, skin infection, cholelithiasis, urolithiasis, and fracture of a large bone. The incidence of any psychiatric diagnosis in the 14–90 days after COVID‐19 diagnosis was 18.1%, including 5.8% that was the first diagnosis. The first diagnosis of dementia in the 14–90 days after COVID‐19 diagnosis was 1.6% in people 65 and older. A psychiatric diagnosis in the previous year was associated with a higher incidence of COVID‐19 diagnosis (relative risk 1.65). This risk was independent of known COVID‐19 physical health risk factors, but residual confounding by socioeconomic factors cannot be excluded.^[^ ^85^ ^]^

Stroke is on the verge of the brain and vascular diseases, as vascular etiology is the basis of brain damage. However, stroke is typically treated by neurologists and is classified as a brain disease. The risk of thrombosis in the cerebral vessels (as well as in other organs) appears to be higher with COVID‐19 infection and outweighs that seen with other viral infections, such as influenza. A retrospective cohort study conducted at two academic hospitals in New York City included adult patients with emergency department visits or hospitalizations for COVID‐19 from March 4, 2020, through May 2, 2020. Among 1916 patients with emergency department visits or hospitalizations with COVID‐19, 31 (1.6%) had an acute ischemic stroke. The median age of patients with stroke was 69 years; 18 (58%) were male. The stroke was the reason for hospital admission in 8 cases (26%). In comparison, 3 of 1486 patients with influenza (0.2%) had an acute ischemic stroke. Adjusted for age, sex, and race, the likelihood of stroke was higher with COVID‐19 infection than with influenza infection (odds ratio, 7.6).^[^ ^86^ ^]^ Also, the proportion of COVID‐19 patients with stroke (1.8%) and in‐hospital mortality (34.4) was exceptionally high. Stroke is relatively frequent and has devastating consequences at any age. The interplay of older age, comorbidities, and severity of COVID‐19 respiratory symptoms is associated with extremely elevated mortality.^[^ ^87^ ^]^

In summary, there is clear evidence of a brain tropism for the SARS‐CoV‐2 virus. This was linked to some very alarming facts related to neurodegeneration, cognitive decline, mental problems, and susceptibility to devastating stroke. Therefore, many neurological and psychiatric conditions can be expected in convalescents, which can profoundly impact our society.

### 4.6 Ears and Eyes

There are close anatomical and functional connections between the eyes, ears, and the brain, so it is not apparent whether the abnormalities should still be attributed to the brain or to these organs. But for the record, we will describe them separately from the brain. There is disturbing evidence that more than 1 in 10 COVID‐19 adults report a change in their hearing status when questioned eight weeks after discharge from the hospital.^[^ ^88^ ^]^ Sudden, irreversible hearing loss has also been reported post‐COVID‐19.^[^ ^89^ ^]^ COVID‐19 also exacerbated tinnitus in 40% of respondents in an online survey of 3103 participants from 48 countries. There are also reports of de novo initiation of tinnitus after COVID‐19.^[^ ^90^ ^]^ In the eyes of all subjects in one study (health‐care professionals) who had recently recovered from severe COVID‐19, hyper‐reflective lesions were seen at the level of ganglion cells and inner plexiform layers, more prominent at the papillomacular bundle in both eyes. No other ocular manifestations were observed, and acuity was normal. These findings might be related to the described symptoms of CNS.^[^ ^91^ ^]^

### 4.7 Other Organs

Hepatic dysfunction has been observed in 14–53% of COVID‐19 patients, particularly in those with serious disease. There have also been reports of acute liver injury associated with higher mortality. Hepatic pathology in COVID‐19 could be related to the virus's cytopathic effect, immune response, or drug‐induced injury.^[^ ^92^ ^]^ SARS‐CoV‐2 also affects the digestive system, causing diarrhea, nausea, vomiting, and diminished appetite. More serious symptoms such as damage to the esophageal mucosa and dilatation, and stenosis of the small intestine have also been described.^[^ ^93^ ^]^ COVID‐19 has also been linked to acute pancreatitis^[^ ^94^ ^]^ and subacute thyroiditis.^[^ ^95^ ^]^ There were also reported cutaneous signs of COVID‐19.^[^ ^96^ ^]^

## 5 Comorbidities and Pre‐Existing Conditions

The contribution of comorbidities to morbidity and mortality has become a hallmark of COVID‐19. It is strongly associated with metabolic and brain disorders, while immune‐related diseases have a relatively weak association with the severity of the COVID‐19 course.

### 5.1 Metabolic Disorders

Diabetes is an independent risk of death from COVID‐19, though the humoral response to SARS‐CoV‐2 was not influenced by glucose levels in the blood and was comparable to nondiabetic patients. It allows for optimism regarding the efficacy of future SARS‐CoV‐2 vaccines in patients with diabetes.^[^ ^97^ ^]^ A multicenter, retrospective cohort study of 1461 COVID‐19 patients admitted to the ICU in 21 centers in Europe, Israel, and the USA, between 02/19/2020 and 05/19/2020, showed a significant relationship between body mass index (BMI) and mechanical ventilation: odds ratio 1.27 per 5 kg m^−2^. The adjusted Cox proportional hazard regression model revealed a significant association between BMI and mortality, which was only increased in obesity class III (≥40 kg m^−2^). In conclusion, in critically ill COVID‐19 patients, a linear association between BMI and the need for mechanical ventilation was observed. This association was independent of other metabolic risk factors and more pronounced in younger females. There was a nonlinear association between BMI and mortality risk.^[^ ^98^ ^]^ Another study also shows obesity above a BMI of 40, to be associated with a significant increase in deaths in a cohort of 6916 COVID‐19 patients from a large integrated health‐care organization located in southern CA.^[^ ^99^ ^]^ Hypertension is one of the most frequent causes of increased risk for hospitalization^[^ ^100^ ^]^ and mortality from COVID‐19.^[^ ^101^ ^]^

### 5.2 Brain Disorders

Patients with a pre‐existing psychiatric condition experience higher mortality from COVID‐19. This trend was also seen earlier with other infections. It is intriguing that patients with psychiatric disorders also have a shorter life expectancy (reduction by ten years). The causes are unknown, but they may be associated with a general increased inflammatory status, which on the one hand, affects the brain function and, on the other hand, exacerbates the symptoms of infections. There is also a possibility that psychotropic drugs have a sensitizing effect on the body for infections.^[^ ^102^ ^]^

### 5.3 Immune‐Related Conditions

Surprisingly, human immunodeficiency virus (HIV) does not affect the course of COVID‐19. While a weaker immune system may facilitate the entry of SARS‐CoV‐2 into the body, it may also blunt autoreaction of the immune system, which may reduce the severity of the disease.^[^ ^103^ ^]^ Similarly, the course of COVID‐19 is not worse in immunosuppressed patients, which may be associated with the same mechanisms as in the case of HIV.^[^ ^104^ ^]^ However, virus shedding in immunocompromised patients might be prolonged.^[^ ^105^ ^]^ There have also been relatively infrequent hospital admissions of COVID‐19 patients with asthma.^[^ ^106^ ^]^ Multiple sclerosis drugs, which are often immunosuppressive, are also neutral to the course of COVID‐19 disease.^[^ ^107^ ^]^

### 5.4 Other Comorbidities

While obstructive sleep apnea (OSA) does not affect the risk of contracting COVID‐19, it worsens the clinical course according to a study based on Finnish national health registries and the FinnGen Study cohort. OSA patients were hospitalized 5 times more frequently, irrespective of age, sex, BMI, hypertension, diabetes, coronary heart disease, asthma, and chronic obstructive pulmonary disease. Therefore, OSA should be considered as a risk factor for COVID‐19‐related hospitalization. Notably, this effect cannot be explained by other comorbidities.^[^ ^108^ ^]^ We hypothesize that air circulation abnormalities in patients suffering from OSA may facilitate the ingress of viral particles through the mucous membranes.

Vertebral fractures (V.F.s) can manifest both patient's metabolic status and may directly impact cardiorespiratory function. These fractures are present in 36% of COVID‐19 patients who undergo a lateral chest X‐ray in the emergency department. Patients with V.F.s were hospitalized more frequently (88% vs 74%), required mechanical ventilation at a higher rate (49% vs 29%), and had higher mortality (22% vs 10%), although only the middle variable is statistically significant. While there are several confounding factors, such as age, hypertension, and cardiovascular disease, the presence of V.F.s on admission X‐ray should trigger a worse prognosis alarm.^[^ ^109^ ^]^

Vitamin D deficiency (blood level < 30 ng mL^−1^) doubled mortality among hospitalized patients from 9.7% to 20% in Tabriz, Iran.^[^ ^110^ ^]^ However, vitamin D should rather be used as an indicator of patients’ metabolic status, and its supplementation may not produce positive therapeutic effects.

Inhibition of gastric acid release is a systematic therapeutic strategy used by many individuals suffering from peptic ulcer. Therefore, any potential negative impact of these drugs on outcomes of patients with SARS‐CoV‐2 is important. In the large health‐care system cohort in TN, USA, there was no increase in 30 day mortality due to Famotidine's use in 7158 hospitalized COVID‐19 patients.^[^ ^111^ ^]^ By contrast, a large meta‐analysis showed that proton pump inhibitors exacerbate the course of COVID‐19. Although the effect size was small, clinicians should still perform a careful risk–benefit assessment.^[^ ^112^ ^]^

## 6 Therapeutic Agents against COVID‐19

**Figure 3:**
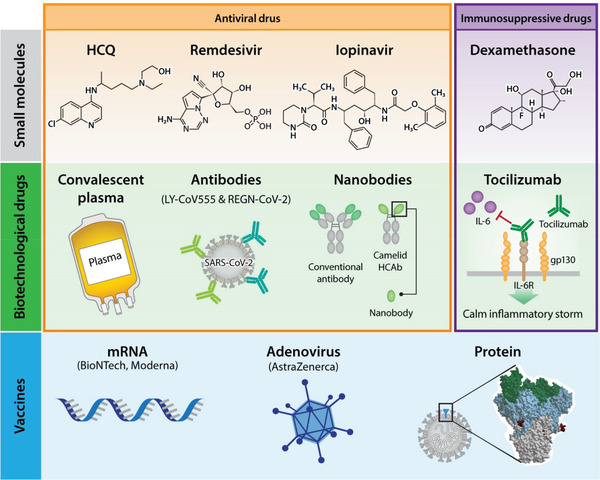
Therapeutic approaches for COVID‐19.

The tremendous impact of COVID‐19 has triggered an enormous effort to find antidotes to SARS‐CoV‐2. A variety of approaches, including small molecules and biotechnological drugs have been thoroughly investigated (**Figure** **3**).

### 6.1 Small Molecules

Low cost, stability, and widespread biodistribution in the body are the advantages of small molecules, but side effects are frequently observed. Hydroxychloroquine (HCQ) is a well‐known drug used in the prophylaxis of malaria and autoimmune diseases. It is relatively safe, although the adverse effects are quite burdensome. An analysis of the initial five randomized controlled clinical trials with 5577 recruited patients demonstrated that HCQ given for pre‐exposure prophylaxis, postexposure prophylaxis, or outpatient therapy is associated with a 24% reduction in COVID‐19 infections, hospitalization, and deaths. No severe cardiac incidents were reported, and most side effects were related to the gastrointestinal system.^[^ ^113^ ^]^ However, a double‐blinded randomized clinical trial revealed that once or twice, weekly HCQ failed to reduce the incidence of COVID‐19 in 1483 high‐risk health‐care workers.^[^ ^114^ ^]^ Another placebo‐controlled study confirmed no preventive value of HCQ in health‐care workers when administered orally at a daily dose of 600 mg for 8 weeks.^[^ ^115^ ^]^ Therefore, the preventive role of HCQ was not accepted.

There is also a group of drugs developed for antiviral therapies, but they were not widely used for their primary purpose for several reasons. One of these drugs, remdesivir, has been shown to positively affect the length of hospitalization in severely ill patients without affecting mortality, as shown in a large, double‐blind, randomized study.^[^ ^116^ ^]^ However, daily remdesivir for 10 days in moderate COVID‐19 did not affect patients’ status at day 11, and the shorter regimen (5 days) affected but of uncertain clinical importance.^[^ ^117^ ^]^ Therefore, remdesivir does not seem to be the ultimate solution for COVID‐19 therapy.

Disappointingly, the SOLIDARITY trial performed on 11 330 patients from 405 hospitals across all 6 World Health Organization regions demonstrated that none of the repurposed antiviral drugs: remdesivir, hydroxychloroquine, lopinavir (fixed‐dose combination with ritonavir), and interferon‐β1a (mainly subcutaneous; initially with lopinavir, later monotherapy) has a positive effect on mortality, initiation of mechanical ventilation, and duration of hospital stay in patients hospitalized for COVID‐19.^[^ ^118^ ^]^

By contrast, systemic corticosteroids are associated with a lower 28 day all‐cause mortality in critically ill COVID‐19 patients and have become the standard of care.^[^ ^119^ ^]^

### 6.2 Biotechnological Drugs

A preliminary study demonstrated very encouraging convalescent plasma therapy results with neutralizing antibody titers above 1:640 in 10 patients. It contributed to the rapid neutralization of viremia and the improvement of blood oxygenation and clinical symptoms in severely ill COVID‐19 patients.^[^ ^120^ ^]^ A review study summarizing the first 27 patients reported across 5 studies revealed that convalescent plasma appears safe, is clinically useful, and reduces mortality.^[^ ^121^ ^]^

However, a randomized clinical trial with the same neutralizing antibody with activity at 1:640 yielded no statistically significant results. Still, the study was terminated prematurely after recruiting 103 patients (half of the planned 200 patients) due to the containment of COVID‐19 in Wuhan.^[^ ^119^ ^]^ Another study using the more liberal 1:160 antibody neutralization guidelines found at 6 weeks after COVID‐19 diagnosis that 70% of patients showed plasma neutralization activity against SARS‐CoV‐2 and only 25% against its S glycoprotein. These numbers decreased by 20–30% in the next 4 weeks (10 weeks after diagnosis). Therefore, it points out that convalescent plasma should be obtained from donors as soon as possible to maximize its efficacy. It is also wake‐up information for vaccine developers that antiviral protection may be subsiding relatively fast.^[^ ^122^ ^]^ Consequently, no therapeutic effects were observed in the study where the vast majority of convalescent plasma donors (94%) had mild disease with a low titer of neutralizing antibodies (up to 1:80, with a median 1:40 in 2/3 of donors, and lower than 1:20 in 1/3 of them).^[^ ^123^ ^]^ Thus, there is currently no evidence of a positive effect of convalescent plasma therapy, but more robustly planned studies may bring more favorable results in future.^[^ ^124^ ^]^

Antibodies are quite specific molecules, and we are now observing an avalanche of antibody‐based therapeutic agents for a variety of diseases. Attempts have also been made to use them in COVID‐19 therapy. With a significant reported increase of IL‐6 in severely ill COVID‐19 patients, this target has been exploited therapeutically. Initial studies revealed encouraging results from the use of an anti‐IL‐6 antibody (tocilizumab) in COVID‐19 therapy. However, no improvement over usual care was found in a randomized clinical trial based on adults hospitalized with COVID‐19 with moderate to severe pneumonia requiring at least 3 L min^−1^ of oxygen but without ventilation or admission to the intensive care unit.^[^ ^125^ ^]^

LY‐CoV555, a rapidly isolated potent neutralizing antibody, is protected in a SARS‐CoV‐2 infection model in nonhuman primates.^[^ ^126^ ^]^ However, an interim analysis of the SARS‐CoV‐2 neutralizing antibody LY‐CoV555 in outpatients with COVID‐19 surprisingly revealed that only a medium dose resulted in eliminating viral load as well as slightly reduced the severity of symptoms and decreased hospitalization rate from 6.3% in the placebo group to 1.6% in patients receiving LY‐CoV555.^[^ ^127^ ^]^ Ly‐CoV555, which is now known as bamlanivimab, has received Food and Drug Administration emergency use authorization to treat recently diagnosed mild to moderate COVID‐19 in high‐risk patients. Although, the clinical trial with the administration of LY‐CoV555 has been halted at the recommendation from the independent Data and Safety Monitoring Board.

REGN‐COV2 is an antibody cocktail that has been shown to be effective in treating SARS‐CoV‐2 in a primate model.^[^ ^128^ ^]^ Reduction of viral levels and improved symptoms in nonhospitalized COVID‐19 patients has also been reported in a press release. Administration of REGN‐COV2 to hospitalized patients with high oxygen therapy requirements has been halted, while the enrollment of patients with no or low oxygen was continued.

Many other potential therapeutic agents are also under development. The Pittsburgh group constructed multivalent nanobodies that achieved ultrahigh neutralization potency (inhibitory concentration 50s as low as 0.058 ng mL^−1^) and could prevent mutational escape. These thermostable molecules can be rapidly produced in bulk and were shown to be functional following lyophilization and aerosolization.^[^ ^129^ ^]^ Mesenchymal stem cells constitute another approach for severely ill patients with COVID‐19 due to their immunomodulatory properties.^[^ ^130^ ^]^

## 7 Vaccines

Vaccines have eradicated or dramatically decreased the incidence of many infectious diseases, improving health and quality of life worldwide. Therefore, it is no surprise that there is a lot of hope and effort involved in developing the COVID‐19 vaccine. There are various molecular approaches to induce immunity against SARS‐CoV‐2 (Figure 3).

We are currently witnessing excellent progress with the two messenger ribonucleic acid (mRNA)‐based vaccines manufactured by Moderna^[^ ^131^ ^]^ and Pfizer/BioNTech.^[^ ^132^ ^]^ Surprisingly, a vaccine based on this technology has never been licensed before, so the challenge is huge. However, mRNA has several very encouraging properties, such as pure chemical production, eliminating the need for xenomaterial, and contamination with proteins or other molecules. It is also relatively easy to make changes to follow the potential genetic drift of the virus. Genetic engineering based on mRNA is characterized by a short‐expression time of the introduced vaccination antigens, contributing to the overall excellent safety profile. The mRNA production method is also fast and scalable, making it well suited to dealing with a rapidly evolving pandemic. Overall, mRNA vaccines have been hailed as a new era in vaccinology.^[^ ^133^ ^]^ Moreover, clinical trials’ results indicate that mRNA‐based vaccines for COVID‐19 are effective more than 90%.^[^ ^134^ ^]^ The third COVID‐19 vaccine, which is the most advanced in development (AZD1222, AstraZeneca), is based on adenoviruses’ older technology.^[^ ^135^ ^]^ It had more serious adverse effects but is still considered a very important candidate for market use. Progress has also been achieved with the Adsorbed COVID‐19 (inactivated) vaccine manufactured by Sinovac – PROFISCOV.^[^ ^136^ ^]^

There are also several very interesting vaccines at the earlier stages of development. The lower‐cost option suitable for developing countries is vaccination using modified receptor‐binding domain (RBD) fragment of spike protein produced in yeasts and administered in a hydrogel medium. It induced a satisfactory titer of antibodies in a mouse model.^[^ ^137^ ^]^ A chimpanzee‐adenovirus‐vectored vaccine encoding a prefusion‐stabilized spike protein (ChAd–SARS‐CoV‐2–S) has advantages for potential intranasal administration.^[^ ^138^ ^]^

While there is a lot of enthusiasm with COVID‐19 vaccines, disadvantages should also be considered. Large and appropriate numbers of patients are enrolled in the vaccination trials; however, it should be emphasized that the studies are performed over a very short period of months, while typical vaccines are rolled out over several years. Therefore, data on both long‐term safety and efficacy lack in this context and worrying observation declined in the level of SARS‐CoV‐2 antibodies in convalescents.^[^ ^122^ ^]^ There was a report showing a fast decrease of IgM and IgA class antibodies against SARS‐CoV‐2. By contrast, the neutralizing IgG‐based antibodies against the RBD of spike protein decreased more slowly but steadily over 90 days in most hospitalized COVID‐19 patients.^[^ ^139^ ^]^ A similar decrease in neutralizing activity from 6% to 4.4% (26.5% drop) was also observed in the UK population study of 365 104 participants, where a lateral flow immunoassay test for IgG was self‐administered.^[^ ^140^ ^]^ However, anti‐SARS‐CoV‐2 antibodies have been demonstrated not only in the serum but also in saliva.^[^ ^141^ ^]^ There is anecdotal evidence of a protective role of Bacille Calmette‐Guérin vaccination in middle‐aged individuals who were vaccinated as infants, but this was dismissed in a natural experiment conducted in Sweden.^[^ ^142^ ^]^

## 8 Long‐Term Consequences of COVID‐19

### 8.1 Persistence of Symptoms

**Figure 4:**
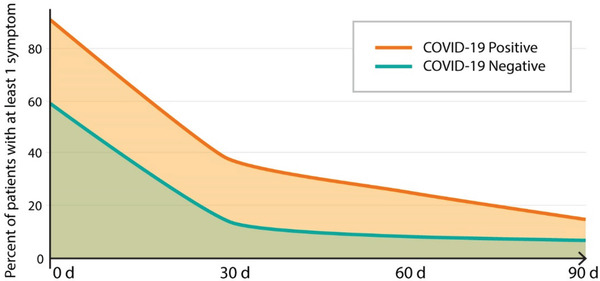
Lingering effects of COVID‐19 – the higher number of patients with COVID‐19, comparing to patients seen for other than COVID‐19 purposes, experienced at least one symptom over extended period of time.

The persistence of symptoms seems particularly alarming in the context of the COVID‐19 pandemic. An online survey of 15 763 Helix DNA Discovery Project participants and 5596 Healthy Nevada Project participants provided a fascinating comparison of the occurrence of new symptoms in a virus‐untested population and patients who survived COVID‐19. It has been demonstrated that 43.4% of COVID‐19 patients have symptoms lasting longer than 30 days, and 24.1% had at least one symptom at the 90 day time point. These numbers were higher for patients with more severe disease: 59.4% at 30 days and 40.6% at 90 days. Besides, even among asymptomatic and very mild cases, 14.3% of patients experienced complications that persisted for 30 days or longer (**Figure** **4**). Consequently, all COVID‐19 positive groups had more persistent symptoms than participants in the general untested population (8.6%).^[^ ^143^ ^]^

Follow up of 108 patients in Malaga (Spain) 12 weeks after acute SARS‐CoV‐2 infection revealed lingering symptoms in most patients (75.9%), with dyspnea being the most frequent. Younger age (<65 years) and the health‐care profession were risk factors for the persistence of symptoms. Anti‐SARS‐CoV‐2 antibodies were detected in all patients.^[^ ^144^ ^]^

A UK study followed 58 COVID‐19 patients post‐hospital discharge and 30 comorbidity‐matched controls for 2–3 months not only by physical exam but also a series of objective assessments such as multiorgan MRI, spirometry 6 min walk test, cardiopulmonary exercise test, quality of life, cognitive and mental health assessments. Persistent dyspnoea and severe fatigue were observed in over half of the patients. This coincided with frequent changes on MRI scans (60% in lungs, 29% in kidneys, 26% in heart, 10% in liver, along with abnormalities observed in central regions of the brain). Cognitive performance, mental health, exercise tolerance, and quality of life were also significantly impaired in patients hospitalized for COVID‐19. Serum markers of inflammation correlated with imaging, clinical, and functional changes.^[^ ^145^ ^]^

### 8.2 Multisystem Inflammatory Syndrome (MISC)

The multisystem inflammatory syndrome is a new pediatric disease associated with SARS‐CoV‐2. It typically occurs long after initial infection with SARS‐CoV‐2 (symptomatic or asymptomatic), and the disease is dangerous and potentially lethal. With prompt diagnosis and medical attention, most children will survive, but the long‐term effects of the condition are presently unknown.^[^ ^146^ ^]^ The literature search yielded 39 observational studies (*n* = 662 patients). While 71.0% of children (*n* = 470) were admitted to the intensive care unit, only 11 deaths (1.7%) were reported. The average length of hospital stay was 7.9 days. The most common clinical symptoms were fever (100%, *n* = 662), abdominal pain or diarrhea (73.7%, *n* = 488), and vomiting (68.3%, *n* = 452). Serum inflammatory, coagulative, and cardiac markers were considerably abnormal. Mechanical ventilation and extracorporeal membrane oxygenation were necessary in 22.2% (*n* = 147) and 4.4% (*n* = 29) of patients, respectively. An abnormal echocardiography was observed in 314 of 581 individuals (54.0%) with depressed ejection fraction (45.1%, *n* = 262 of 581) comprising the most common aberrancy.^[^ ^147^ ^]^ There is also one report of a pediatric patient with fatal MISC with detected SARS‐CoV‐2 in cardiac tissue. It indicates that muscles, including the heart, maybe directly infected with the virus, and this condition frequently characterized by fulminant immune activation can be lethal.^[^ ^148^ ^]^

### 8.3 Reinfections

Reinfections are another troubling phenomenon, especially if they occur within a short period. Considering that lingering symptoms are observed in many patients, and there is also relatively permanent damage observed in some patients, there is a risk that each reinfection could lead to accumulating injury, subsequently leading to death. In many circumstances, it is difficult to provide solid evidence of reinfection instead of the persistence of the disease. However, several studies have reported cases with convincing evidence of reinfection each time with the different genetic variants. For example, a reinfection with the new D614G variant has been shown. The antibody waned relatively quickly after the first infection. Reinfection did not generate new B cell clones, so it is likely that the immune response was still based on the previous infection. Reinfection was also associated with milder symptoms in this case. The genetic drift of viruses must be considered while developing vaccines and immunological drugs.^[^ ^149^ ^]^ In the other two cases, also proven by distinct genetic variants, reinfections were by far more severe than the initial ones^[^ ^150^, ^151^ ^]^ In summary, reports of reinfections in literature are scarce, thus it seems not be a major threat to the society at least as of now.

## 9 Mortality

The COVID‐19 deaths are devastating, although they need to be put in the context of the all‐cause mortality to understand the pandemic's real impact on society. It has been shown that in countries with moderate COVID‐19 mortality, such as Australia or Canada, the pandemic has practically no impact on all‐cause mortality. By contrast, in territories with the highest COVID‐19 mortality rates, such as the USA and the European Union, all‐cause mortality increases dramatically. It has a profound impact on society's welfare.^[^ ^152^ ^]^ The excess of all‐cause mortality beyond COVID‐19 deaths may be due in part to delay in seeking medical care, such as the decrease in hospital admissions for transient ischemic attack, mild and moderate stroke during the COVID‐19 era as a consequence. It may be due to patients’ fear of seeking help and contributing to increased all‐cause mortality.^[^ ^153^ ^]^

As of October 15, there have been 216 025 deaths in the United States from COVID‐19. Overall, it is estimated that there were 299 028 excess deaths in the United States between late January and October 3.^[^ ^154^ ^]^ Excess deaths in New York City during the peak of the 1918 H1N1 influenza pandemic were compared with those during the initial COVID‐19 outbreak.^[^ ^155^ ^]^ During the March–April period, 8369 deaths were expected in New York City, while a staggering 29 703 deaths were recorded there, so the mortality rate has tripled.^[^ ^156^ ^]^ The risk of death is also 5 times higher in patients hospitalized with COVID‐19 than with contemporary flu.^[^ ^157^ ^]^ It should also be emphasized that resource constraints and the depletion of reserves in overcrowded hospitals are particularly responsible for sudden mortality rates.^[^ ^158^ ^]^ Despite the odds, a single health system study in the USA revealed a gradual improvement in risk‐adjusted mortality rates, which may be a combination of the growing use of new pharmacological regimens and raising experience in handling patients, lower viral load exposures due to community awareness, social distancing, and mask wearing.^[^ ^159^ ^]^ The same trend was observed in the UK.^[^ ^160^ ^]^

## 10 A Summary and an Outlook for Future Perspectives

The COVID‐19 menace is felt at every step. Though, the world learns fast and implements countermeasures. Pandemic management takes place on many levels, and it is expected to start diminishing the COVID‐19 impact over time. Public health plays a critical role. We are also witnessing spectacular successes of medical research. Paradigm‐shifting vaccines introduced at crazy speeds are safe and very effective.^[^ ^161^ ^]^ It was not only related to well established science behind them, but also broad stream of money behind it, as well as enormous effort of regulatory bodies and volunteers to make it possible. The antibodies against COVID‐19 are preventing hospitalizations in vulnerable groups of patients.^[^ ^162^ ^]^ We are also witnessing positive developments dedicated to seriously ill patients, such as corticosteroids. Once the acute phase of a pandemic is taken under control, there will be a dire need to redirect resources to target the lingering effects of COVID‐19. Stem‐cell‐based regenerative medicine may play a vital role in rebuilding damaged tissues.^[^ ^163^ ^]^ The mental health consequences of COVID‐19 may require various interventions at the community level.^[^ ^164^ ^]^ In summary, the COVID‐19 menace seems to be gradually grasped by humankind, but we are still far from a return to normal.

## Conflict of Interest

The authors declare no conflict of interest.
